# Correlation between human papillomavirus infection and bladder transitional cell carcinoma

**DOI:** 10.1186/1471-2334-5-102

**Published:** 2005-11-08

**Authors:** MR Barghi, A Hajimohammadmehdiarbab, SMM Hosseini Moghaddam, B Kazemi

**Affiliations:** 1 Assistant Professor Of Urology, Shohada Tajrish Hospital, Shahid Beheshti University Of Medical Sciences, Urology and Nephrology Research Center, Tehran, Iran; 2 Resident Of Urology, Shohada Tajrish Hospital, Shaeed Beheshti University Of Medical Sciences, Urology and Nephrology Research Center, Tehran, Iran; 3 Research Consultant, Assistant Professor Of Infectious Diseases And Tropical Medicine, Master of Public Health, Urology and Nephrology Research Center, Shaheed Beheshti University Of Medical Sciences, Tehran, Iran; 4 Cellular and Molecular Biology Research Center, Urology and Nephrology Research Center, Shaheed Beheshti University of Medical Sciences, Urology and Nephrology Research Center, Tehran, Iran; 5 Urology and Nephrology Research Center (UNRC), Shohada Tajrish Hospital, Shaheed Beheshti University of Medical Science, Tehran Iran

## Abstract

**Background:**

To determine the association of human papillomavirus infection (HPV) and transitional cell carcinoma (TCC).

**Methods:**

Using polymerase chain reaction, fifty-nine bladder tissue specimens of patients with transitional cell carcinoma of bladder compared with 20 bladder samples of cases with non-neoplastic disorders.

**Results:**

Male to female ratio was similar in the two groups (50/9 vs. 16/4, *P *= 0.62). Mean age was 67 ± 10.8 years and 52 ± 20.3 years in the case and control groups, respectively (*P *= 0.6). Of the 59 tissue specimens with diagnosis of transitional cell carcinoma, HPV DNA was detected in 21 (35.6%) samples, while it was present in only one sample (5%) in the control group (*P *= 0.008). HPV18 was the most common type of virus with the incidence rate of 17/21(81%).

**Conclusion:**

HPV might play a causative role in transitional cell carcinoma of bladder in our geographic area.

## Background

The etiology of transitional cell carcinoma (TCC), which represents 90 percent of bladder malignancies, is not quite clear, while squamous cell carcinoma (5%) of the bladder is well associated with some factors like urinary stones and prolonged infections. Some genital malignancies like vulvar or cervical neoplasms are associated with previous infection with human papillomavirus (HPV). Recently, conflicting findings have been reported on association of HPV infection and TCC [1-5].

Using PCR, the overall prevalence of human papillomavirus (HPV) DNA in penile carcinoma is about 40–45%, which is similar to the detection rate of HPV-DNA in vulvar carcinoma [6]. Males may be carriers of oncogenic HPVs and male partners may markedly contribute to the risk of developing cervical cancer in their female partners [7]. HPV detection rate in cases of invasive cervical cancer can be as high as 90 % [8]. Because of vicinity of bladder to mucosal surface of urogenital tract, HPV may play a significant role in TCCs.

While Kamel et al found HPV DNA in 57% of cases of TCC [1], Tekin's study [9] and Mvula's report [10] did not support the etiologic role of HPV in bladder carcinogenesis. In the present study, we compared the rate of HPV positivity in formalin-fixed, paraffin-embedded tissues from the urinary bladder of patients with transitional cell carcinomas and non-neoplastic bladder tissue samples. To our knowledge, this is one of limited number of studies that used non-neoplastic bladder tissue specimens as controls.

## Methods

The medical records and bladder tissue specimens of 59 consecutive patients with transitional cell carcinoma of bladder who underwent transurethral resection of bladder tissue from October1999 to May 2002 were reviewed and compared with those of 20 consecutive patients with non-neoplastic disorders who were served as controls. So, a total of 79 biopsy tissues of urinary bladder were re-evaluated and analyzed by polymerase chain reaction (PCR). The designing and conducting of this study was in accordance with the ethical standards of UNRC ethics committee. Table 1 demonstrates histopathological diagnosis of specimens in the control group in detail. Since there are infrequent indications for bladder tissue sampling in non-neoplastic conditions, comparing to malignant lesions, we found only 20 non-neoplastic bladder tissue specimens.

**Table 1:** histologic examination of tissue specimens from control group

**Finding**	**Frequency**
Inflammatory tissue (non specific cystitis)	5
Granuloma formation	1
Eosinophilic cystitis	2
Squamous metaplasia	1
Fibrosis	3
Hemorrhagic cystitis	1
Severe chronic cystitis + Atypical epithelial hyperplasia	1
Transitional papilloma	
Chronic cystitis	1
Glandular cystitis + squamous metaplasia	2
Fibromuscular tissue	1
Normal mucosa	1
	1

### Sample

Specimens were taken from paraffin-embeded bladder tissues stored in laboratory of our hospital- Tajrish Shohada medical center. All specimens were collected and transported to molecular biology research center of shaheed Beheshti University of Medical Sciences under a well-controlled condition. Biopsy specimens were digested in lyses buffer (0.33 M sucrose, 10 mM Tris bas, 5 mM MgCl2, 2% Titon X-100) at 37°C for 4 hours. Phenol-chloroform extraction and ethanol precipitation were performed. The precipitated DNA was suspended in distilled water and used for amplification.

### Primers

Two primer pairs were designed of HPV L1 gene (encodes the major capsid protein): one primer pair amplify a 269-bp fragment of HPV 6, 11, 31 and 33, and another primer pair amplify a 564-bp fragment of HPV 16, 18 and 35; and HPV types were defined by PCR products restriction analysis. PCR: Amplifications were carried out in 50-μl volumes. The reaction mixture contained 1.5 mM MgCl_2_, 0.1 mM of each dNTP, 20 pico mol of each primer, 0.1 μg DNA, 1.25 unit of Taq DAN polymerase and 1× PCR buffer. PCR conditions are: 30 cycles of denaturing at 94°C for 30 sec., annealing temperature at 45°C for 30 sec. and extension at 72°C for 30 sec. PCR reaction was performed using automated thermal cycler machine (model personal, Eppendorf Co, Germany).

### Electrophoresis

PCR products were electrophoresis on 2% agarose gel. Gels were stained by ethidium bromide and photographed by UV transilluminator at 254 nm.

Statistical analysis was performed using Mann-Whitney, chi-square and fisher's exact tests as appropriate with significance considered at *P *< 0.05.

## Results

The study population comprised 59 cases and 20 controls whose demographic and clinical characteristics are given in table 2. There was no difference in male to female ratio in the two groups (50/9 vs. 16/4, *P *= 0.62). Mean age was 67 ± 10.8 (range 38 to 85) years and 52 ± 20.3 (range18 to 81) years in the case and control groups, respectively (*P *= 0.6).

**Table 2:** demographic and clinical characteristics

	**Case**	**Control**
**Age**	67 ± 10.8(38–85)	52 ± 20.3(18–81)

**Sex**		
Male	50(84.7%)	16(80%)
Female	9(15.3%)	4(20%)

**UII**	3(5.1%)	0

**Metastasis**		
Bone	2	-
Lung	2	-
Local lymph nodes	1	-
Both liver & kidney	2	-

**Site of lesion in bladder**		
Dome	6	-
Left lateral	21	-
Right lateral	14	-
Bilateral	10	-
Trigone	1	-
Trigone & right lateral	2	-
Bladder neck	2	-
Diffuse	2	-
Ureteral orifice	1	-

**Other simultaneous problems**		
Hypertension	7(11.8%)	2(10%)
IHD	5(8.5%)	0
CLL	0	1(5%)
CRF	0	1(5%)
DM	6(10.1%)	0
Asthma	1(1.7%)	0
Hepatitis	1(1.7%)	0
Renal Stone	3(5%)	0
Tuberculosis	1(1.7%)	0
Hyperthyroidism	2(3.4%)	1(5%)
Neurogenic tumor	0	1(5%)
Urethral stricture	0	1(5%)

**Number of tumor lesions in cystoscopy**		
1	26(44.1%)	-
2	9(15.3%)	-
3	2(3.4%)	-
4	3(5.1%)	-
>4	19(32.2%)	-

Of the 59 tissue specimens with diagnosis of transitional cell carcinoma, HPV DNA was detected in 21 (35.6%) samples. Human papillomavirus DNA was positive in only one sample (5%) in the control group (*P *= 0.008) that was histolologically diagnosed as severe chronic cystitis with some degrees of atypical hyperplasia, that might be considered as a pre-malignant condition.

Type specific primers also analyzed the positive samples. In the case group, sequences of HPV 6 genome were detected in 2/21 patients (9.5%), one male and 1 female; HPV 18 was found in 14/21 patients (66.7%),13 males and 1 female ; and HPV 33 sequences were detected in2/21(9.5%), 1 male and 1 female. In 3 HPV-positive male cases (14.3%) more than one type was found; 2 patients with HPV6/HPV18, and 1 witht HPV18/HPV33. Thus, HPV18 was the most common type of virus with the incidence rate of 17/21(81%). HPV18 was also found in one male patient in the control group. HPV types 11, 16, 31 were present in no tissue specimens. Table 3 shows HPV types in two groups, distinctly.

The histopathological stages and grades of tissue samples with diagnosis of transitional cell carcinoma considering infection with HPV are given in Table 4. No significant correlation existed between tumor stages and presence of HPV. Additionally, there was no significant difference between low-grade (grade 1 and 2) and high grade (grade 3) tumors with regard to HPV infection (36% Vs.33.3%, *P *= 0.59).

**Table 3:** positive samples analyzed by type specific-primers

**HPV-type/Group**	**HPV6**	**HPV18**	**HPV33**	**HPV6/HPV18**	**HPV18/HPV33**
**Case**	2(9.5%)	14(66.7%)	2(9.5%)	2(9.5%)	1(4.8%)
**Control**	-	1(100%)	-	-	-

**Table 4:** distribution of tumor stage and grade among cases.

**Stage or grade/HPV infection**	**Stage**							**Grade**		
	
	**T_is_**	**T_a_**	**T_1_**	**T_2a_**	**T_2b_**	**T_3_**	**T_4_**	**I**	**II**	**III**
**infected**	1 (4.8%)	5 (23.8%)	12 (57.1%)	3 (14.3%)	0	0	0	8 (38.1%)	10 (47.6%)	3 (14.3%)
**Non infected**	0	20 (52.6%)	12 (31.6%)	5 (13.1%)	1 (2.6%)	0	0	13 (34.2%)	19 (50%)	6 (15.8%)

Smoking rate in the case group was significantly higher than control group (54.2% vs 10%, *P *= 0.00). In the case group, 12 out of 21 (57%) infected patients and 20 out of 38 (52.6%) non-infected were cigarette smokers, respectively (*P *= 0.7). Ten cases (16.9%) but no patient in the control group were addicted to opium (*P *= 0.05). On the other hand, four infected (18.2%) and 6 non-infected (10.5%) subjects were addicted to opium (*P *= 0.36).

## Discussion

One of the most common malignancies, especially in developing countries, is transitional cell carcinoma of the bladder. Several chemical agents have been suspected to have a role in its development. HPV plays an etiological role for genital tumors, but the exact effect of this virus in transitional cell carcinoma of bladder is still vague. More than a decade ago, a report of HPV-16 positive bladder carcinoma in a patient with attenuated natural killer cell function and aplastic anemia was published [11]. In the same year, another paper briefed a transplant patient with bladder tumor in whom HPV 11 was detected [12]. Agliano and colleagues investigated the presence of HPV types 16 and 18 DNA in formalin-fixed, paraffin-embedded tissues from the urinary bladder (46 transitional cell carcinomas and 10 non-neoplastic normal urinary samples) of non-immune deficient cases. HPV16 and/or HPV18 genomes were detected in 23 of 46 (50%) bladder carcinomas and in none of 10 (0%) non-neoplastic urinary samples[13]. In 1995, Kamel et al. analyzed 47 bladder carcinomas for the presence of DNA-HPV subtypes 6, 11, 16, 18, 31 and 33 by nucleic acid in situ hybridization. HPV DNA was found in 27/47 (57%) bladder carcinomas, with multiple subtypes in 20 cases (76). To our knowledge, our study has one of the largest control groups of non-neoplastic bladder tissue specimens. The high prevalence of HPV infection in the present study demonstrates an association between HPV and TCC of the bladder.

Human papillomavirus seems to be related to the etiology of bladder tumor because of its high prevalence in samples obtained from bladder tumors in the present study and some previous researches. Our study demonstrated HPV-positivity rate in 35.6% of cases and 5% of controls. It seems that carcinoma development may be triggered by HPV infection. Inactivation of the tumor suppressor pRB by the human papillomavirus (HPV) oncoprotein E7 is a mechanism by which HPV promotes cell growth [14]. Human papillomavirus type 16 proteins, E6 and E7, have been shown to cause centrosome amplification and lagging chromosomes during mitosis, leading to chromosomal instability. Genomic instability is thought to be an essential part of the conversion of a normal cell to a cancer cell [15] Kamel and colleagues demonstrated concurrent HPV positivity and abnormal p53 protein accumulation in 18 out of 47 cases, 14 showing the presence of HPV subtypes 16 and/or 18 DNA[1]. In our study, HPV 18 was the most frequent type(81%) requiring more specific epidemiologic studies and experimental investigations.

HPV may have a great role in progression of TCCs toward higher stages and/or grades by inactivation of the tumor suppressors or other unknown mechanisms.

Larue et al. reported that sensitivity of detection of HPV is largely dependent on a series of technical factors such as tissue fixation, DNA preparation and amplification conditions. In their study, presence of HPV correlated with grade but not stage of the tumors. [16]. Our specimens were formalin-fixed, paraffin-embedded tissues from the urinary bladder. Using fresh or frozen tumor material will probably increase the sensitivity. The high incidence demonstrated in the present study might be higher if fresh specimens were available and used in order to overcome the probability of qualitative loss of DNA in the material.

High rate of HPV positivity in present study suggests that other sexually transmissible viruses may play some roles in development and progression of transitional cell carcinoma of the bladder. As Gazzaniga et al demonstrated, there may be a high synergism between Human Papillomaviruses type 16 and 18 (HPV 16, HPV 18), Epstein-Barr virus (EBV), cytomegalovirus (CMV) and herpes simplex virus type 2 (HSV-2) and bladder carcinogenesis[17]. In this regard, further investigation with a large number of patients sounds to be required.

On the basis of high rate of HPV positivity in TCC cases (35.6%), comparing with control group(5%), the present report supports an etiologic role of HPV in bladder carcinogenesis.

## Conclusion

This quasi-experimental study is the first report from Iran on HPV infection and transitional cell carcinoma. Our findings, in spite of some previous reports, suggest that HPV may play a causative role in transitional cell carcinoma of bladder in our geographic area. Public education regarding HPV transmission and high-risk behavior may be helpful for decreasing the incidence of urogenital malignancies.

## Abbreviations

HPV: Human Papilloma Virus

## Competing interests

The author(s) declare that they have no competing interest.

## Authors' contributions

M.R.B. and A.H. introduced the cases and performed surgical operations. S.M.M.H.M. designed the study and performed statistical analyses. B. K. performed laboratory examinations and PCR. All authors read and approved the final manuscript.

## Pre-publication history

The pre-publication history for this paper can be accessed here:


